# Supplementary material for: Modeling glioblastoma heterogeneity as a dynamic network of cell states
Source: Mol Syst Biol. 2021 Sep 16;17(9):e10105. doi: 10.15252/msb.202010105 (PMC8444284; doi:10.15252/msb.202010105)
Supplement: Supplementary file 6 — Source Data for Figure 5 [file MSB-17-e10105-s004.zip › Figure5A_sourcedata/GSEA_3071/hallmarks_stateIII.GseaPreranked.1623742540963/HALLMARK_BILE_ACID_METABOLISM.html]

Details for gene set HALLMARK\_BILE\_ACID\_METABOLISM[GSEA]

|  || Dataset | state33071 |
| Phenotype | NoPhenotypeAvailable |
| Upregulated in class | na\_neg |
| GeneSet | HALLMARK\_BILE\_ACID\_METABOLISM |
| Enrichment Score (ES) | -0.36036378 |
| Normalized Enrichment Score (NES) | -1.4406 |
| Nominal p-value | 0.086513996 |
| FDR q-value | 0.19880806 |
| FWER p-Value | 0.98 |
Table: GSEA Results Summary

  

Fig 1: Enrichment plot: HALLMARK\_BILE\_ACID\_METABOLISM      
 Profile of the Running ES Score & Positions of GeneSet Members on the Rank Ordered List

  

| PROBE | GENE SYMBOL | GENE\_TITLE | RANK IN GENE LIST | RANK METRIC SCORE | RUNNING ES | CORE ENRICHMENT || 1 | OPTN |  |  | 296 | 0.292 | -0.0367 | No |
| 2 | SOD1 |  |  | 315 | 0.287 | -0.0028 | No |
| 3 | DHCR24 |  |  | 598 | -0.260 | -0.0402 | No |
| 4 | FADS2 |  |  | 1437 | -0.302 | -0.2140 | No |
| 5 | PECR |  |  | 1854 | -0.327 | -0.2765 | No |
| 6 | EFHC1 |  |  | 1946 | -0.332 | -0.2553 | No |
| 7 | PEX6 |  |  | 2288 | -0.356 | -0.2949 | No |
| 8 | PEX7 |  |  | 2545 | -0.377 | -0.3099 | Yes |
| 9 | FADS1 |  |  | 2588 | -0.381 | -0.2697 | Yes |
| 10 | PEX11A |  |  | 2909 | -0.409 | -0.2968 | Yes |
| 11 | SLC22A18 |  |  | 2971 | -0.416 | -0.2567 | Yes |
| 12 | NUDT12 |  |  | 2997 | -0.420 | -0.2069 | Yes |
| 13 | ACSL1 |  |  | 3221 | -0.446 | -0.2042 | Yes |
| 14 | MLYCD |  |  | 3224 | -0.447 | -0.1449 | Yes |
| 15 | RETSAT |  |  | 3245 | -0.450 | -0.0898 | Yes |
| 16 | ABCA5 |  |  | 3377 | -0.475 | -0.0597 | Yes |
| 17 | ABCA1 |  |  | 3421 | -0.485 | -0.0058 | Yes |
| 18 | SLC29A1 |  |  | 3473 | -0.499 | 0.0480 | Yes |
| 19 | DIO2 |  |  | 3510 | -0.511 | 0.1071 | Yes |
Table: GSEA details [plain text format]

  

Fig 2: HALLMARK\_BILE\_ACID\_METABOLISM: Random ES distribution      
 Gene set null distribution of ES for **HALLMARK\_BILE\_ACID\_METABOLISM**
